# Autoimmune blistering skin diseases triggered by COVID-19 vaccinations: An Australian case series

**DOI:** 10.3389/fmed.2022.1117176

**Published:** 2023-01-10

**Authors:** Timothy L. Cowan, Cheng Huang, Dédée F. Murrell

**Affiliations:** ^1^Department of Dermatology, St. George Hospital, Sydney, NSW, Australia; ^2^Faculty of Medicine, University of New South Wales, Sydney, NSW, Australia

**Keywords:** pemphigoid, pemphigus, autoimmune blistering, COVID-19, vaccination, flare

## Abstract

Autoimmune blistering skin diseases (AIBD) can be induced or flared by a multitude of sources, however, there have been some reports suggesting that this occurrence is due to COVID-19 vaccinations. At a single academic blistering disease centre in Sydney, Australia, a retrospective review was conducted, identifying 59 patients with AIBD seen between February 2021 and November 2022. Secondary to recent COVID-19 vaccination, four patients had induction of bullous pemphigoid, three patients had a flare of pre-existing bullous pemphigoid, one patient had induction of pemphigus, and two patients had a flare of pre-existing pemphigus vulgaris. This adds to our understanding of the role of vaccinations in the activity of AIBD.

## Introduction

Autoimmune blistering skin diseases (AIBD), which can be categorised into both pemphigoid and pemphigus, are a potentially life-threatening group of diseases that occur due to the development of autoantibodies against proteins in the skin and mucous membranes. AIBDs can be induced and worsen in activity from a variety of causes, of which vaccinations had been reported prior to the COVID-19 pandemic (1–5). A reported mechanism behind this is the disruption of the basement membrane architecture that results in the production of anti-basement membrane antibodies with secondary AIBD in some people (2). Flares and induction of AIBD have been reported secondary to immunisations for swine flu, herpes zoster, and combined vaccines for diphtheria, pertussis, tetanus, poliomyelitis, and hepatitis B (1–4). Widespread vaccination for COVID-19 has made a major impact on patients with AIBD (6). Since the rollout of the COVID-19 vaccinations, there have been a small number of reports of the induction of new-onset AIBD and flares of pre-existing AIBD and only rare reports of an association with pemphigus (7–9).

## Materials and methods

We retrospectively identified patients with autoimmune blistering diseases (AIBD) from an academic blistering disease clinic in Sydney, Australia from February 2021 to November 2022. Patients were included if they had a flare of a pre-existing AIBD, or if they had a new AIBD induced by a COVID-19 vaccine. Patient demographics and scores of disease activity including Bullous Pemphigoid Disease Area Index (BPDAI) (10) and Pemphigus Disease Area Index (PDAI) (11) before and after COVID-19 vaccination were collected.

## Results

During the data collection, 29 patients with bullous pemphigoid (BP) and 30 with pemphigus were identified. New-onset BP induced by a COVID-19 vaccination was identified in four cases (four of 59; 6.8%, Table 1). Of these, the median age was 67 years and three (three of four, 75%) were male. The median time from their last COVID-19 vaccination to symptom onset was 29 days (5–123). All four patients had two doses of the AstraZeneca vaccination prior to BP onset. One patient had the BioNTech Pfizer booster prior to the BP onset and one had an additional flare after a Moderna booster. A 49-year-old female patient was reported to have new-onset pemphigus induced by a third BioNTech Pfizer COVID-19 vaccination (Table 1).

**TABLE 1:** Demographic information, summary of vaccinations, and scores of autoimmune blistering skin diseases (AIBD) activity.

	Number	Age	Sex	COVID-19 vaccination prior to AIBD	Time from last vaccination to symptom onset (days)	BPDAI/PDAI prior to vaccination (for pre-existing AIBD)	BPDAI/PDAI post-vaccination at time of flare/Disease onset
Drug-induced BP	1	82	M	AZ	31	–	19
	2	62	M	Pfizer	123	–	47
	3	71	M	AZ	26	–	78
	4	60	F	AZ	5	–	5
Flare of BP	5	82	M	AZ	92	14	47
	6	83	M	Pfizer	90	0	20
	7	86	F	Pfizer	21	0	4
Drug-induced pemphigus	8	49	F	Pfizer	92	–	69
Flare of pemphigus	9	32	F	Pfizer	6	0	4
	10	73	M	Pfizer	15	0	6

Five patients had a flare of a pre-existing AIBD due to a COVID-19 vaccination (five of 59, 8.5%), including three cases of BP and two cases of pemphigus. The median age was 82 years and three (three of five, 60%) were male. The median time from the last COVID-19 vaccination to the disease flare was 21 (6–92) days. Two (two of five, 40%) had the BioNTech Pfizer vaccination and three (three of five, 60%) had the AstraZeneca vaccination. Four of these patients (four of five, 60%) subsequently had a BioNTech Pfizer booster prior to a further disease flare.

## Discussion

This case series provides further evidence of the impact of COVID-19 vaccinations on AIBD. Prior to the COVID-19 pandemic, there were several reports of the induction and flaring of AIBD due to non-COVID-19 vaccinations (1–5). Since the COVID-19 pandemic, there has been prompt and widespread vaccination against COVID-19. In conjunction with the roll-out of these vaccines, there have been reports of new and flaring pre-existing cases of AIBD (6–9).

**FIGURE 1:**
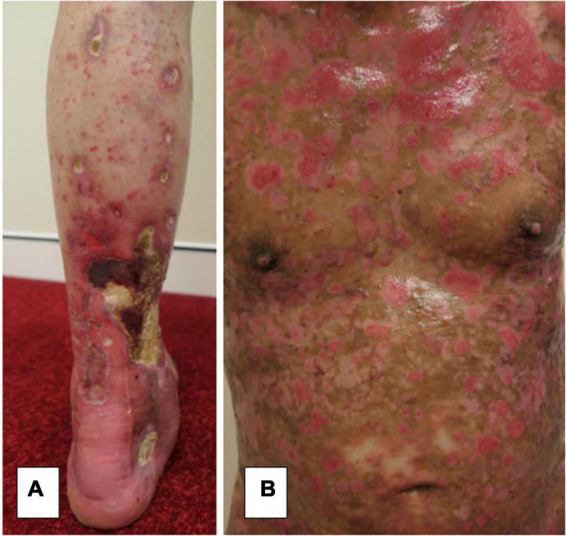
**(A)** Severe lower limb necrotic ulceration secondary to IgA vasculitis from severe bullous pemphigoid induced by a COVID-19 vaccination with **(B)** extensive activity and damage scores.

A systematic review has reported this occurrence more often with bullous pemphigoid than pemphigus and occurring between 1 day and 6 weeks post-vaccination (6). This review reports only 11 cases of pemphigus triggered by COVID-19 vaccination. In other reports, COVID-19 vaccinations have been linked to the induction of severe disease in the case of bullous pemphigoid (7, 8). One case in our series had induction of severe bullous pemphigoid with a BPDAI of 78 (Table 1) after the AstraZeneca COVID-19 vaccine (8). This required approximately 6 months of admission in hospital and respite facilities and was complicated by IgA vasculitis, a large lower limb necrotic ulceration, and *Staphylococcus aureus* septicaemia (Figure 1).

**FIGURE 2:**
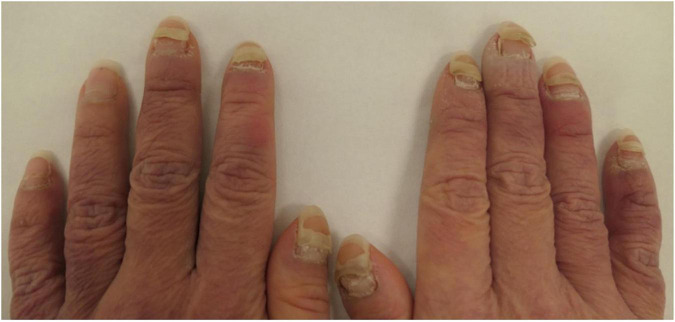
Severe pemphigus vulgaris induced by a COVID-19 vaccination with extensive onycholysis.

Our series adds further cases of the inducing and flaring of bullous pemphigoid secondary to recent COVID-19 vaccinations. To add to a rarely reported phenomenon, we also report one case of pemphigus vulgaris induced by COVID-19 vaccination and two cases of pemphigus vulgaris that have flared secondary to COVID-19 vaccination. These cases of flared pemphigus had achieved control of disease activity with a PDAI score of zero prior to flares triggered by COVID-19 vaccination (Table 1). The case of pemphigus induced by a COVID-19 vaccination had severe pemphigus vegetans with an initial PDAI 69 and severe and extensive onycholysis (Figure 2).

It is evident that COVID-19 vaccinations contribute to flares of AIBD as well as the induction of the disease. This evidence clarifies the importance of including a history of recent vaccinations, including COVID-19, in the assessment of patients with the new AIBD activity.

## Data availability statement

The raw data supporting the conclusions of this article will be made available by the authors, without undue reservation.

## Ethics statement

Written informed consent was obtained from the individual(s) for the publication of any potentially identifiable images or data included in this article.

## Author contributions

DM contributed to the conception and design of the study. CH organised the database, data entry, statistical analysis, and results and wrote the initial abstract. All authors wrote sections of the manuscript, contributed to the manuscript, and also revised, read, and approved the submitted version.
